# Efficacy and safety of immune checkpoint inhibitors in advanced biliary tract cancer: a real-world study

**DOI:** 10.3389/fimmu.2025.1493234

**Published:** 2025-03-31

**Authors:** Yichen Zheng, Jiamin Guo, Tonghui Ren, Ji Ma, Dan Cao

**Affiliations:** Department of Medical Oncology, Cancer Center and Laboratory of Molecular Targeted Therapy in Oncology, West China Hospital, Sichuan University, Chengdu, Sichuan, China

**Keywords:** biliary tract cancer, immune checkpoint inhibitors, real-world study, efficacy, safety, first-line, second or later lines

## Abstract

**Background:**

Immune checkpoint inhibitors (ICIs) combined with gemcitabine and cisplatin chemotherapy have become the standard first-line treatment for advanced biliary tract cancer (BTC). However, real-world evidence on domestic ICIs widely used in China and the therapeutic outcomes across treatment lines remains limited. This study aimed to assess the real-world effectiveness and safety profiles of ICIs in advanced BTC patients, while concurrently elucidating potential efficacy variations among distinct ICI subtypes.

**Methods:**

We analyzed patients with unresectable, locally advanced, or metastatic BTC treated with ICIs at West China Hospital (January 2019–October 2023). Primary endpoint was overall survival (OS), while secondary endpoints included progression-free survival (PFS), objective response rate (ORR), disease control rate (DCR), and safety. Kaplan-Meier survival curves, propensity score matching (PSM), and Cox proportional hazards regression analyzed treatment efficacy.

**Results:**

A total of 221 advanced BTC patients were enrolled. Among them, 137 patients received ICIs treatment in the first line, while 84 patients in the second or later lines. For patients treated with ICIs as first-line therapy, the median OS was 15.7 months (95% CI: 13.1-19.8) and PFS was 8.4 months (95% CI: 7.6-10.3). In contrast, patients treated in second or later lines had shorter median OS of 9.8 months (95% CI: 8.1–12.3) and median PFS of 5.6 months (95% CI: 4.2–6.8). The reduced efficacy in later-line treatments may reflect prior therapeutic resistance and generally poorer patient conditions compared to first-line recipients. 211 (95.5%) patients experienced at least one adverse event (AE), and 93 (42.1%) of them experienced grade 3 or higher AEs. The incidence of immune-related adverse events (irAEs) was 35.8%, with 8.6% of patients experiencing grade 3-4 irAEs. The most common ICI treatments are with Durvalumab or Sintilimab, which we are interested in comparing. Durvalumab showed numerically superior OS vs Sintilimab (19.3 vs 10.2 months, p<0.001) in unmatched analysis, though significance attenuated after PSM (16.1 vs 13.1 months, p=0.299).

**Conclusion:**

ICIs demonstrate robust efficacy and manageable toxicity in real-world settings, supporting their use in both first- and later-line treatments for advanced BTC. However, whether domestic ICI alternatives remain viable options warranting further validation.

## 1 Introduction

Biliary tract cancer (BTC) includes intrahepatic cholangiocarcinoma (ICC), perihilar cholangiocarcinoma, distal cholangiocarcinoma, and gallbladder cancer (1). Compared to other gastrointestinal tumors, BTC is relatively rare. However, its incidence is increasing globally (1). Due to late diagnosis, high tumor aggressiveness, and limited effective treatment options, the prognosis of BTC is generally poor (2). For localized disease, surgical resection remains the only potentially curative method, but the postoperative recurrence rate is as high as 70%-75% (3). Additionally, many patients are diagnosed at an advanced stage, limiting surgical treatment options. According to the ABC-02 trial results, gemcitabine combined with cisplatin chemotherapy was the main treatment for locally advanced or metastatic BTC, but the efficacy was not ideal (4). Over the following decade, many attempts were made to improve efficacy, such as using novel drugs or adding a third chemotherapeutic agent to the cisplatin-gemcitabine (CisGem) regimen, but unfortunately, clinical improvements were not significant (5).

In recent years, immune checkpoint inhibitors (ICIs) have been rapidly changing the treatment paradigm across various cancer types. However, BTC is typically considered an immunologically “cold” tumor (6–8), and thus the clinical efficacy of ICIs in BTC has generally been disappointing. With the exception of select patient subgroups exhibiting high microsatellite instability (MSI-H)/mismatch repair deficiency (dMMR) or high PD-L1 expression (9), the effectiveness of ICIs remains limited. Data from various small single-arm studies indicate that, for the broader BTC patient population, ICIs yield ORR ranging from 3% to 13% and median OS ranging from 5.2 to 8.1 months (10, 11). Only with the recent publication of the TOPAZ-1 and KEYNOTE-966 trials have ICIs been formally incorporated into first-line treatment for advanced BTC. Specifically, the TOPAZ-1 trial demonstrated that durvalumab combined with CisGem significantly improved median OS (12.8 vs. 11.5 months) and PFS (7.2 vs. 5.7 months) compared to chemotherapy alone (12). Similarly, the KEYNOTE-966 study indicated that pembrolizumab combined with CisGem significantly extended median OS (12.7 vs. 10.9 months) and PFS (6.5 vs. 5.6 months) (13). Nevertheless, these survival gains have been modest. Therefore, the overall efficacy of ICIs in advanced BTC warrants further exploration in real-world settings.

However, treatment options remain very limited for advanced BTC that has failed first-line treatment. Although the ABC-06 study evaluated FOLFOX (folinic acid, fluorouracil, and oxaliplatin) as a second-line treatment after CisGem progression, the efficacy was low, and chemotherapy alone could not meet clinical needs (14). Furthermore, several promising targets have been identified in BTC, including fibroblast growth factor receptor 2 (FGFR-2) fusions/rearrangements, isocitrate dehydrogenase 1/2 (IDH-1/2) mutations, human epidermal growth factor receptor 2 (HER-2) amplification, B-Raf proto-oncogene serine/threonine kinase (BRAF) V600E mutation, and neurotrophic tyrosine receptor kinase (NTRK) fusions. Targeted therapies against these targets have shown promising results in some phase II studies (15). Accumulated clinical evidence has suggested that systemic treatment with tyrosine kinase inhibitors (TKIs) combined with immunotherapy may improve clinical outcomes in advanced BTC patients who have failed first-line treatment (16, 17). Nevertheless, the value of immunotherapy in the later lines for advanced BTC still lacks high-quality evidence and remains in the clinical exploration stage.

Although pembrolizumab and Durvalumab are recommended as the standard first-line immunotherapeutic agents for advanced BTC, their high cost and lack of health insurance coverage make them difficult choices for many patients in China. Some domestic PD-1 or PD-L1, due to their lower cost and higher accessibility, are more widely used among Chinese patients, but their specific efficacy has not been evaluated. Although several real-world studies have assessed the efficacy of these ICIs in advanced BTC (18, 19), the limited sample size and different research focus indicated the need for more evidence. In summary, there is currently a lack of data on the efficacy and safety of ICIs treatment for advanced BTC patients (including first-line or ≥2 lines), as well as an analysis of the differences among various ICIs drugs.

Therefore, we conducted this single-center real-world study with a large sample size and detailed subgroup analysis to reflect the safety and efficacy of ICIs in different treatment stages of advanced BTC.

## 2 Methods

### 2.1 Study design

**Figure 1:**
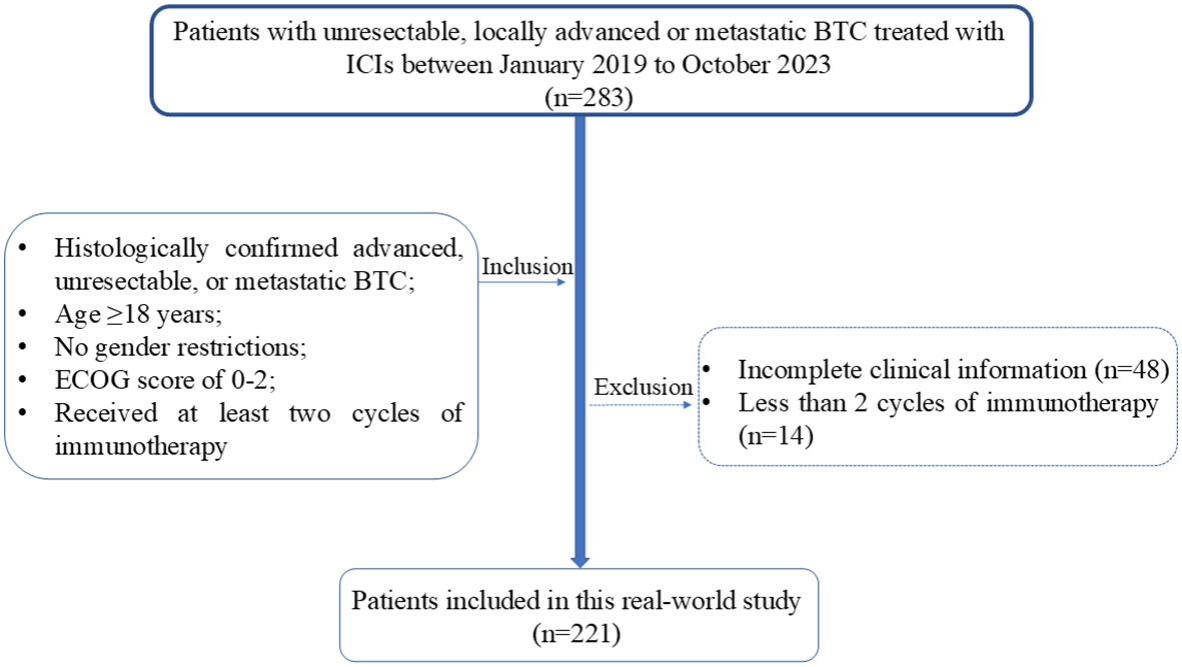
Patients inclusion and exclusion flowchart. BTC. BTC, Biliary Tract Cancer; ECOG, Eastern Cooperative Oncology Group; ICIs, Immune Checkpoint Inhibitors.

This study is a retrospective single-center analysis of real-world data. The study population included patients with unresectable, locally advanced, or metastatic BTC who received ICIs treatment at West China hospital between January 2019 and October 2023. Inclusion criteria were as follows: histologically confirmed advanced, unresectable, or metastatic BTC; age ≥18 years; any gender; ECOG performance status of 0-1; and at least two cycles of immunotherapy, whether as monotherapy or in combination with chemotherapy. Patients with incomplete clinical information or without pathological evidence of BTC were excluded (**Figure 1**). We collected clinical characteristics, laboratory and imaging reports, treatment history, survival status, treatment-related adverse events (AEs), and immune-related adverse events (irAEs) through electronic medical records and telephone follow-ups. The follow-up cutoff date was April 30, 2024.

### 2.2 Treatment protocol

The treatment regimens and dosages of ICIs, chemotherapeutic drugs, and targeted therapy drugs in this study were determined by oncologists. The initial doses were based on the guidelines of the National Comprehensive Cancer Network (NCCN) or the Chinese Society of Clinical Oncology (CSCO). The most common ICIs included Durvalumab 1500 mg every three weeks, Sintilimab, pembrolizumab, and camrelizumab 200 mg every three weeks, and toripalimab 240 mg every three weeks. Only a minority of patients received alternative ICIs such as Nivolumab and Tislelizumab. The doses of chemotherapeutic and targeted drugs were adjusted during treatment based on patient tolerance and general condition.

### 2.3 Evaluation

Baseline CT or MRI scans were performed before starting ICIs treatment, followed by imaging assessments every 2-3 cycles. Tumor efficacy was evaluated according to the Response Evaluation Criteria in Solid Tumors (RECIST 1.1) (20). The primary endpoint of this study was overall survival (OS), and secondary endpoints included progression-free survival (PFS), objective response rate (ORR), disease control rate (DCR), and safety. OS was defined as the time from initiation of ICIs treatment to death caused by cancer. PFS was defined as the time from initiation of ICIs treatment to disease progression, death, or the last follow-up date (whichever occurred first). Patients lost to follow-up were right censored at the last contact date. ORR was defined as the proportion of patients achieving complete response (CR) or partial response (PR) according to RECIST 1.1 criteria. DCR was defined as the proportion of patients achieving CR, PR, and stable disease (SD). Safety assessment included the occurrence of AEs from the start of ICIs treatment to the last follow-up. This included irAEs (mainly rash, thyroid-related events, immune-related pneumonitis, immune-related myocarditis, etc.) and other adverse events. The severity of AEs was graded according to the Common Terminology Criteria for Adverse Events (CTCAE) version 5.0 (21), and the incidence of irAEs was calculated separately.

### 2.4 Statistical analysis

Quantitative data were analyzed using t-tests or Mann-Whitney U tests (for non-normally distributed data), and qualitative data were analyzed using chi-square test or Fisher’s exact probability method. In survival analysis, hazard ratios (HRs) and their corresponding p-values were calculated using both univariate and multivariate Cox proportional hazards regression models (22). Multivariate cox regression covariates were selected based on univariate analysis (p<0.1). Kaplan-Meier (KM) method was used to plot survival curves (23). The fundamental formula of the Cox proportional hazards regression model is as follows:


$$h(t|X)=h_{0}\left(t\right)\text{exp}\left(\beta_{1}X_{1}+\beta_{2}X_{2}+\cdots +\beta_{p}X_{p}\right)$$

In this equation, *h*(*t*|*X*) represents the instantaneous hazard at time *t*, given the covariates *X*. The term *h*_0_(*t*) is the baseline hazard function, which indicates the hazard when all covariates are zero. The expression $\text{exp}\left(\beta_{1}X_{1}+\beta_{2}X_{2}+\cdots +\beta_{p}X_{p}\right)$ is the linear combination of covariates, reflecting the influence of these covariates on the hazard. Here, *β*_1_,*β*_2_,…*β_p_* are the regression coefficients, quantifying the effect of each covariate on the hazard. Finally, *X*_1_,*X*_2_,…*X_p_* are the covariates, which can be either continuous or categorical variables.

Due to the retrospective nature of this study and the observed baseline differences between patients receiving Durvalumab and Sintilimab treatments, propensity score matching (PSM) was employed (24, 25) to address the limitations of using regression analysis to adjust for potential confounders, particularly when the effective sample size (number of outcome events) is small (26, 27). This approach aimed to minimize confounding factors and enhance comparability between treatment groups, facilitating a more robust evaluation of the efficacy and safety of the two regimens. Propensity scores were calculated using logistic regression, incorporating potential confounders that could influence OS as matching variables. These confounders were identified as factors with a p-value< 0.1 in the univariate Cox proportional hazards regression analysis of OS in the pre-matched sample. The formula for estimating propensity scores using the logistic regression model is as follows:


$$logit\left[P\left(G=1|X\right)\right]=\alpha +\beta_{1}x_{1}+\cdots +\beta_{m}x_{m}$$

In this formula, *G* represents the group or exposure factor, where *G* = 1 indicates the individual is in the exposed group and *G* = 0 indicates the individual is in the control group. *X* is the vector of covariates, *X* = (*x_1_*,*x_2_*,…,*x_m_*), while *P*(*G* = 1*X*) represents the estimated probability of an individual receiving the treatment (*G* = 1) given the covariates *X*, which corresponds to the individual’s propensity score. A 1:2 nearest neighbor matching method with a caliper width of 0.1(the maximum allowable difference in propensity scores) was applied to match individuals based on their propensity scores (24, 25, 28). Although equal ratio matching is sometimes considered more persuasive, the disparity in group sizes justified using a 1:2 matching ratio to better utilize available data. Nevertheless, we conducted a 1:1 matching as a sensitivity analysis. A p-value<0.05 was considered statistically significant. Data analysis and survival curve plotting were performed using R software.

## 3 Results

### 3.1 Patient baseline characteristics

A total of 221 patients with advanced BTC who met the study criteria were included. This cohort comprised 142 cases of intrahepatic cholangiocarcinoma (64.3%), 41 cases of extrahepatic cholangiocarcinoma (18.6%), and 38 cases of gallbladder cancer (17.2%). Among them, there were 117 male patients (52.9%) and 104 female patients (47.1%). Chronic hepatitis B virus infection was present in 152 patients (68.8%). 110 patients (49.8%) had primary unresectable tumors, and 111 patients (50.2%) had postoperative recurrence. Metastatic BTC was present in 80.1% of the patients, while 19.9% had locally advanced BTC. More than half of the patients (62.0%) had poorly differentiated tumors. In the entire cohort, 137 patients (62.0%) received ICIs as first-line treatment, while 84 patients (38.0%) received ICIs as second or later-lines treatment. 148 patients (67.0%) were treated with PD-1 inhibitors, and 73 patients (33.0%) were treated with PD-L1 inhibitors. The most commonly used ICIs included Durvalumab (59 patients, 26.7%), Sintilimab (50 patients, 22.6%), Camrelizumab (25 patients, 11.3%), Pembrolizumab (20 patients, 9.0%) and Toripalimab (20 patients, 9.0%). In addition, 192 patients (86.9%) received chemotherapy combined with immunotherapy, 41 patients (18.6%) received anti-angiogenic therapy, 53 patients (24.0%) received radiotherapy, and 56 patients (25.3%) received local interventional therapy. Baseline sociodemographic and clinical characteristics of all patients are shown in **Table 1**.

**Table 1:** Patients’ characteristics in the entire cohort.

Characteristic	Overall, N = 221*^1^*	First line, N = 137*^1^*	≥2 lines, N = 84*^1^*
Age, years	58.0 (10.3)	57.7 (10.8)	58.4 (9.4)
Sex			
Male	117 (52.9%)	74 (54.0%)	43 (51.2%)
Female	104 (47.1%)	63 (46.0%)	41 (48.8%)
Virology_status			
No viral hepatitis	68 (30.8%)	38 (27.7%)	30 (35.7%)
Any viral hepatitis B	152 (68.8%)	98 (71.5%)	54 (64.3%)
Prior hepatitis C	1 (0.5%)	1 (0.7%)	0 (0%)
Disease_status			
Initially unresectable	110 (49.8%)	85 (62.0%)	25 (29.8%)
Recurrent	111 (50.2%)	52 (38.0%)	59 (70.2%)
Disease_classification			
Locally advanced	44 (19.9%)	30 (21.9%)	14 (16.7%)
Metastatic	177 (80.1%)	107 (78.1%)	70 (83.3%)
Site_of_origin			
Intrahepatic	142 (64.3%)	96 (70.1%)	46 (54.8%)
Extrahepatic	41 (18.6%)	23 (16.8%)	18 (21.4%)
Gallbladder	38 (17.2%)	18 (13.1%)	20 (23.8%)
Degree_of_differentiation			
Poorly	137 (62.0%)	92 (67.2%)	45 (53.6%)
moderately-to-well	84 (38.0%)	45 (32.8%)	39 (46.4%)
Type_of_ICIs			
Anti-PD-1	148 (67.0%)	74 (54.0%)	74 (88.1%)
Anti-PD-L1 = 1	73 (33.0%)	63 (46.0%)	10 (11.9%)
ICIs			
Durvalumab	59 (26.7%)	52 (38.0%)	7 (8.3%)
Sintilimab	50 (22.6%)	21 (15.3%)	29 (34.5%)
Camrelizumab	25 (11.3%)	12 (8.8%)	13 (15.5%)
Pembrolizumab	20 (9.0%)	13 (9.5%)	7 (8.3%)
Toripalimab	20 (9.0%)	6 (4.4%)	14 (16.7%)
others	47 (21.3%)	33 (24.1%)	14 (16.7%)
Combination_with_chemotherapy			
No	29 (13.1%)	4 (2.9%)	25 (29.8%)
Yes	192 (86.9%)	133 (97.1%)	59 (70.2%)
Combination_with_anti_angiogenic_drugs			
No	180 (81.4%)	124 (90.5%)	56 (66.7%)
Yes	41 (18.6%)	13 (9.5%)	28 (33.3%)
ECOG_performance_status			
0	165 (74.7%)	105 (76.6%)	60 (71.4%)
1	55 (24.9%)	32 (23.4%)	23 (27.4%)
2	1 (0.5%)	0 (0%)	1 (1.2%)
Have_received_radiotherapy			
No	168 (76.0%)	110 (80.3%)	58 (69.0%)
Yes	53 (24.0%)	27 (19.7%)	26 (31.0%)
Have_undergone_interventional_therapy			
No	165 (74.7%)	102 (74.5%)	63 (75.0%)
Yes	56 (25.3%)	35 (25.5%)	21 (25.0%)
Pre_treatment_CA199_level_less_than_500 U/mL			
No	65 (29.4%)	38 (27.7%)	27 (32.1%)
Yes	156 (70.6%)	99 (72.3%)	57 (67.9%)
Pre_treatment_CEA_level_less_than_5 ng/mL			
No	82 (37.1%)	49 (35.8%)	33 (39.3%)
Yes	139 (62.9%)	88 (64.2%)	51 (60.7%)
Pre_treatment_CA125_less_than_28.65 U/mL			
No	115 (52.0%)	68 (49.6%)	47 (56.0%)
Yes	106 (48.0%)	69 (50.4%)	37 (44.0%)
NLR_less_than_3			
No	110 (49.8%)	65 (47.4%)	45 (53.6%)
Yes	111 (50.2%)	72 (52.6%)	39 (46.4%)

CA125, Cancer Antigen 125; CA199, Carbohydrate Antigen 19-9; CEA, Carcinoembryonic Antigen; ECOG, Eastern Cooperative Oncology Group; ICI, Immune Checkpoint Inhibitor; NLR, Neutrophil-to-Lymphocyte Ratio.

*^1^* Sample mean (SD), for age characteristic; Sample size n (percent %), for others.

### 3.2 Efficacy

The median follow-up duration for the entire cohort was 10.1 months (95% CI: 9.6-10.7). Among the 221 patients, 2 achieved CR (0.9%), 50 achieved PR (22.6%), 112 achieved SD (50.7%), and 57 did not achieve either response or disease control, experiencing progressive disease (PD) at the time of the first efficacy assessment (25.8%). The ORR and DCR were 23.5% and 74.2%, respectively. In the first-line treatment group, 2 patients achieved CR (1.5%), 40 achieved PR (29.2%), 73 achieved SD (53.3%), and 22 experienced PD (16.1%). The ORR and DCR for first-line patients were 30.7% and 83.9%, respectively. In the second or later-lines treatment group, no patients achieved CR, 10 achieved PR (11.9%), 39 achieved SD (46.4%), and 35 experienced PD (41.7%). The ORR and DCR were 11.9% and 58.3%, respectively (**Table 2**).

**Table 2:** Tumor response in patients treated with ICIs therapy, for overall sample, first-line, and ≥2-lines.

Tumor response	All patients (n=221)	First line (n=137)	≥2 lines (n=84)
CR	2 (0.9%)	2 (1.5%)	0
PR	50 (22.6%)	40 (29.2%)	10 (11.9%)
SD	112 (50.7%)	73 (53.3%)	39 (46.4%)
PD	57 (25.8%)	22 (16.1%)	35 (41.7%)
ORR (CR+PR)	52 (23.5%)	42 (30.7%)	10 (11.9%)
DCR (CR+PR+SD)	164 (74.2%)	115 (83.9%)	49 (58.3%)

CR, Complete Response; PR, Partial Response; SD, Stable Disease; PD, Progressive Disease.

**Figure 2:**
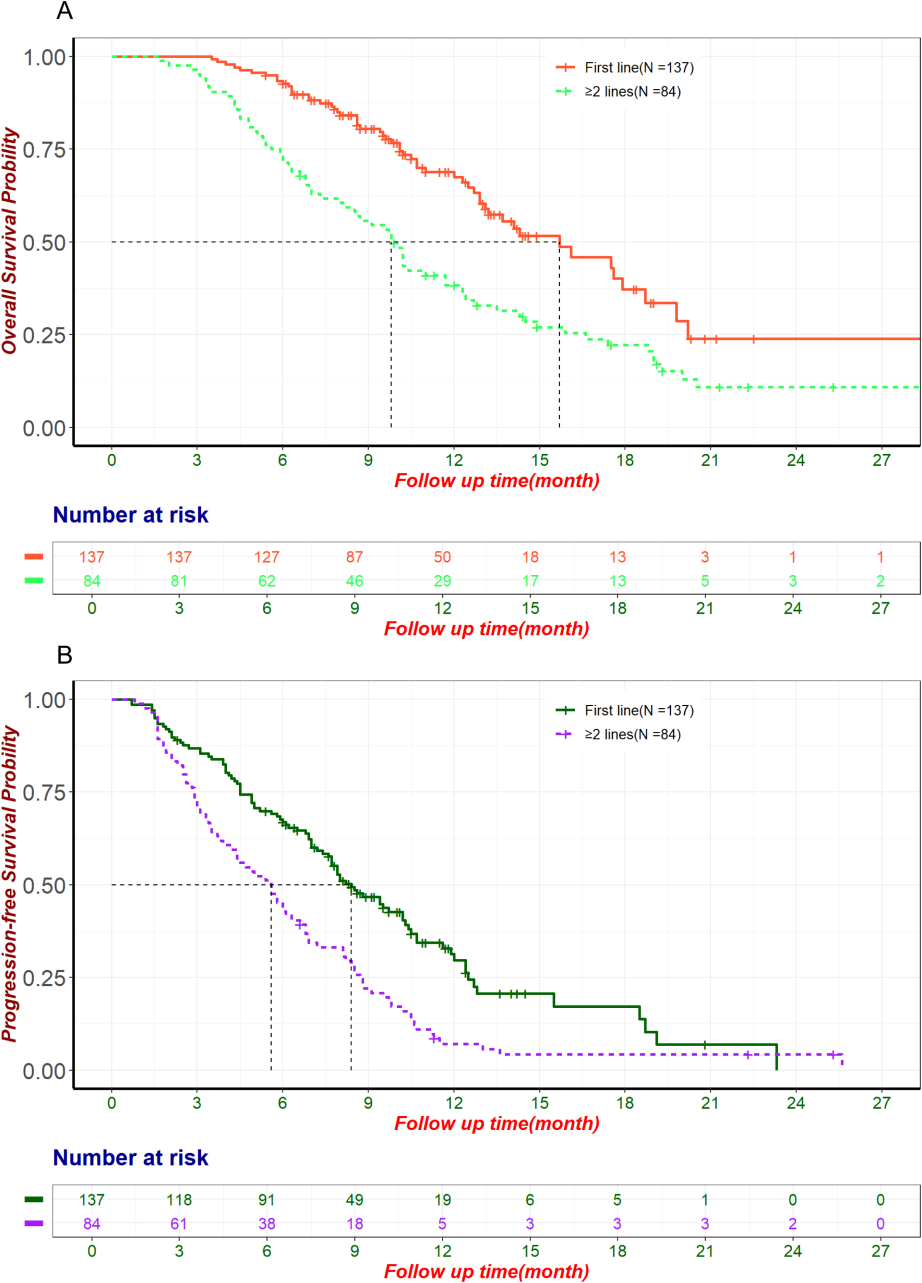
The OS and PFS for first-line and second or later-lines. **(A)** Kaplan-Meier curve of OS in patients treated with ICIs in the first line and second or later-lines. **(B)** Kaplan-Meier curve of PFS in patients treated with ICIs in the first line and second or later-lines. ICIs, Immune Checkpoint Inhibitors; OS, Overall Survival; PFS, Progression-Free Survival.

By the follow-up cutoff date (April 30, 2024), 174 patients experienced disease progression, and 126 of them died. The median OS and PFS for the entire cohort were 12.9 months (95% CI: 11.7-14.9) and 7.2 months (95% CI: 6.3-8.2), respectively. For patients receiving first-line ICIs treatment, the median OS was 15.7 months (95% CI: 13.1-19.8) and PFS was 8.4 months (95% CI: 7.6-10.3). For patients receiving second or later-lines ICIs treatment, the median OS was 9.8 months (95% CI: 8.1-12.3) and PFS was 5.6 months (95% CI: 4.2-6.8). The OS and PFS for first-line and second or later-lines are shown in **Figure 2**. Univariate and multivariate Cox regression analysis (**Table 3**) indicated that tumor differentiation, pre-treatment CA-199 levels<500 U/ml, pre-treatment CA-125 levels<28.65 U/ml, and the number of ICIs treatment lines were independent risk factors for OS (p<0.05). All these factors, together with received subsequent treatments, were the independent predictive factors for PFS (p<0.05) (**Supplementary Table S1**). This suggests a consistent relationship between the two outcome indicators, OS and PFS.

**Table 3:** Univariate and multivariate cox proportional hazards regression for OS in the overall population.

Covariate	All patients (n=221)	HR (univariable)	HR (multivariable)
Sex			
Male	117 (52.9%)		
Female	104 (47.1%)	0.93 (0.65-1.33, p=.698)	
Age (Mean ± SD)	58.0 ± 10.3	1.01 (0.99-1.03, p=.326)	
Virology_status			
No viral hepatitis	68 (30.8%)		
Any viral hepatitis B	152 (68.8%)	0.94 (0.65-1.37, p=.754)	
Prior hepatitis C	1 (0.5%)	3.54 (0.48-26.0, p=.215)	
Disease_status			
Initially unresectable	110 (49.8%)		
Recurrent	111 (50.2%)	1.02 (0.71-1.46, p=.920)	
Disease_classification			
Locally advanced	44 (19.9%)		
Metastatic	177 (80.1%)	1.47 (0.92-2.36, p=.110)	
Site_of_origin			
Intrahepatic	142 (64.3%)		
Extrahepatic	41 (18.6%)	1.63 (1.03-2.57, p=.035) ^*^	1.40 (0.88-2.21, p=.155)
Gallbladder	38 (17.2%)	1.72 (1.13-2.63, p=.012) ^*^	1.02 (0.64-1.63, p=.936)
Degree_of_differentiation			
Poorly	137 (62.0%)		
moderately-to-well	84 (38.0%)	0.65 (0.45-0.94, p=.022) ^*^	0.58 (0.39-0.85, p=.006) ^*^
Type_of_ICIs			
Anti-PD-1	148 (67.0%)		
Anti-PD-L1	73 (33.0%)	0.52 (0.34-0.78, p=.002) ^*^	0.66 (0.41-1.04, p=.075)
Combination_with_chemotherapy			
No	29 (13.1%)		
Yes	192 (86.9%)	0.85 (0.53-1.38, p=.517)	
Combination_with_anti_angiogenic_drugs			
No	180 (81.4%)		
Yes	41 (18.6%)	1.15 (0.75-1.76, p=.528)	
ECOG_performance_status			
0	165 (74.7%)		
≥1	56 (25.3%)	1.23 (0.84-1.81, p=.287)	
Have_received_radiotherapy			
No	168 (76.0%)		
Yes	53 (24.0%)	0.86 (0.57-1.29, p=.467)	
Have_undergone_interventional_therapy			
No	165 (74.7%)		
Yes	56 (25.3%)	0.73 (0.48-1.10, p=.134)	
Occurrence_of_irAE			
No	142 (64.3%)		
Yes	79 (35.7%)	0.88 (0.61-1.27, p=.493)	
Pre_treatment_CA199_level_less_than_500 U/mL			
No	65 (29.4%)		
Yes	156 (70.6%)	0.42 (0.29-0.61, p<.001) ^*^	0.35 (0.23-0.53, p<.001) ^*^
Pre_treatment_CEA_level_less_than_5 ng/mL			
No	82 (37.1%)		
Yes	139 (62.9%)	0.52 (0.37-0.75, p<.001) ^*^	0.75 (0.51-1.11, p=.153)
Pre_treatment_CA125_less_than_28.65 U/mL			
No	115 (52.0%)		
Yes	106 (48.0%)	0.47 (0.32-0.68, p<.001) ^*^	0.57 (0.38-0.85, p=.006) ^*^
NLR_less_than_3			
No	110 (49.8%)		
Yes	111 (50.2%)	0.76 (0.53-1.08, p=.125)	
Received_subsequent_treatment			
No	124 (56.1%)		
Yes	97 (43.9%)	0.89 (0.63-1.28, p=.539)	
Use_of_antibiotics_within_one_month_after_immunotherapy			
No	211 (95.5%)		
Yes	10 (4.5%)	1.69 (0.74-3.86, p=.211)	
Smoking_status			
Never	170 (76.9%)		
Former/Current	51 (23.1%)	0.66 (0.42-1.05, p=.076)	0.85 (0.51-1.39, p=.508)
Line_of_treatment_for_ICIs			
First line	137 (62.0%)		
≥2 lines	84 (38.0%)	2.12 (1.48-3.03, p<.001) *	2.22 (1.47-3.33, p<.001) *

n=221, events=126 (for OS, events refer to the number of deaths caused by cancer).

CA125, Cancer Antigen 125; CA199, Carbohydrate Antigen 19-9; CEA, Carcinoembryonic Antigen; ECOG, Eastern Cooperative Oncology Group; ICIs, Immune Checkpoint Inhibitors; irAE, immune-related Adverse Event; NLR, Neutrophil-to-Lymphocyte Ratio; OS, Overall Survival; PD-1, Programmed Death-1; PD-L1, Programmed Death-Ligand 1.

*P < 0.05.

**Figure 3:**
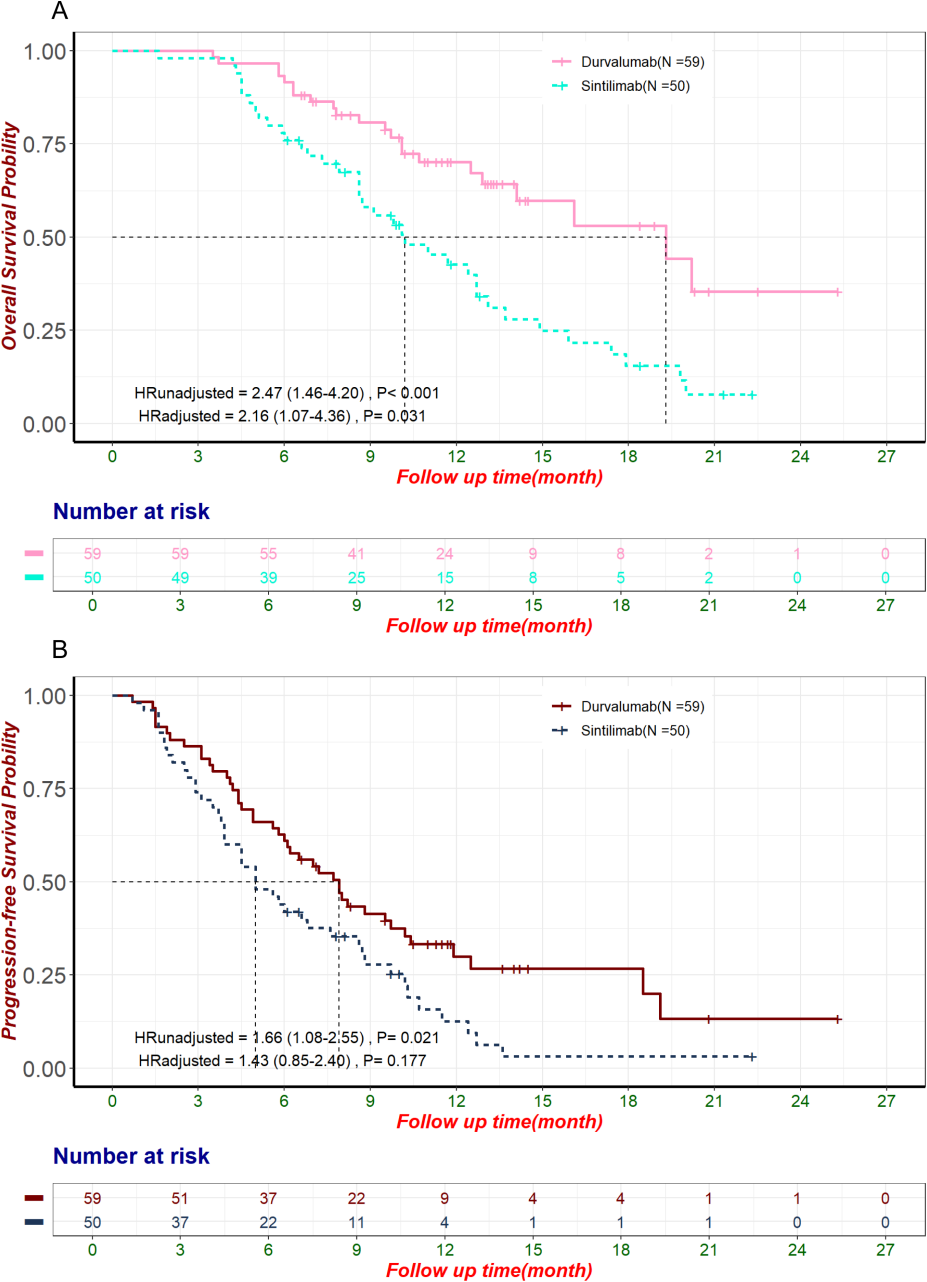
OS and PFS in the pre-matched sample of patients treated with Durvalumab and Sintilimab. HRunadjusted and HRadjusted represent the hazard ratios for Sintilimab compared to Durvalumab, derived from univariate and multivariate Cox proportional hazards regression analyses, respectively. **(A)** Kaplan-Meier curve of OS in the pre-matched sample of patients using Durvalumab and Sintilimab. **(B)** Kaplan-Meier curve of PFS in the pre-matched sample of patients using Durvalumab and Sintilimab. HR, Hazard Ratio; OS, Overall Survival; PFS, Progression-Free Survival.

Among 221 patients, Durvalumab (59 patients, 26.7%) and Sintilimab (50 patients, 22.6%) were the most commonly used ICIs. Significant baseline differences existed between these groups before PSM, including age, disease status, chemotherapy, anti-angiogenic therapy, treatment lines, and CA125 levels (**Supplementary Table S2**). PSM balanced potential confounders that could influence OS (**Supplementary Table S3**), but differences in age, disease status, and anti-angiogenic therapy persisted (**Supplementary Table S4**). In the unmatched cohort, Durvalumab showed superior OS (median: 19.3 months, 95% CI: 14.1–not estimable) vs. Sintilimab (10.2 months, 95% CI: 8.6–13.1; HR: 2.47, 95% CI: 1.46–4.20, p< 0.001; **Figure 3A**). PFS was also longer for Durvalumab (median: 7.9 months, 95% CI: 6.0–10.4) vs. Sintilimab (5.0 months, 95% CI: 3.9–8.6; HR: 1.66, 95% CI: 1.08–2.55, p = 0.021; **Figure 3B**). However, patients in the Durvalumab group had more favorable prognostic factors such as predominantly first-line treatment, necessitating further adjustment for potential biases. After multivariate Cox regression, OS differences remained significant (HR: 2.16, 95% CI: 1.07–4.36, p = 0.031; **Figure 3A**), while PFS differences did not (HR: 1.43, 95% CI: 0.85–2.40, p = 0.177; **Figure 3B**). Given the limitations of regression analysis in small samples, we conducted an exploratory PSM analysis to further control for confounding factors. After PSM (67 matched patients), no significant OS difference was observed (Durvalumab: 16.1 months, 95% CI: 12.5–not estimable; Sintilimab: 13.1 months, 95% CI: 11.0–not estimable; HR: 1.50, 95% CI: 0.70–3.20, p = 0.299; **Supplementary Figure S1**). Multivariate Cox proportional hazards regression in the matched cohort (adjusted HR: 2.08, 95% CI: 0.81–5.37, p = 0.129; **Supplementary Figure S1**) and sensitivity analysis using 1:1 matching ratio (**Supplementary Figure S2**) confirmed this. ORR was higher with Durvalumab (27%) than Sintilimab (14%) in the unmatched cohort, although this difference was not statistically significant (p = 0.094). The observed trend suggests a potential clinical benefit of Durvalumab. This is particularly noteworthy considering that statistical significance (p< 0.05) is relatively difficult to achieve in small-sample comparisons and should be interpreted as a reference tool rather than a definitive measure. Relying solely on p-values may underestimate the actual clinical significance of the observed difference.

The efficacy of other commonly used ICIs is as follows: Camrelizumab (used in 25 patients) demonstrated a median OS of 14.5 months (95% CI: 11.7-not estimable), a median PFS of 7.7 months (95% CI: 6.3-not estimable), and an ORR of 32%. Pembrolizumab (used in 20 patients) had a median OS of 10.2 months (95% CI: 9.6-not estimable), a median PFS of 7.55 months (95% CI: 5.9-18.7), and an ORR of 20%. Toripalimab (used in 20 patients) showed a median OS of 12.3 months (95% CI: 6.3-not estimable), a median PFS of 5.85 months (95% CI: 4.2-9.1), and an ORR of 15%. However, we did not conduct direct comparisons of the efficacy among these ICIs, and the reasons for this will be elaborated in the Discussion section.

### 3.3 Safety

Among the 221 patients receiving ICIs treatment for advanced BTC, 211(95.5%) patients experienced at least one AEs. A total of 93 patients (42.1%) experienced grade 3 or higher AEs. The most common AEs included anemia (40.3%), neutropenia (33.0%), thrombocytopenia (29.0%), and hypoproteinemia (19.9%) (**Table 4**). The most frequent grade 3-4 AEs were neutropenia (13.6%), thrombocytopenia (12.2%), hypoproteinemia (9.5%) and anemia (8.6%). Most patients improved with symptomatic supportive care and/or dose reduction, but 24 patients (10.9%) discontinued treatment due to intolerable AEs. There were no AE-related deaths in the entire cohort.

**Table 4:** Treatment-related adverse events.

Adverse events	All patients (n=221)		First line (n=137)		≥2 lines (n=84)	
	Any grade	Grade 3 or 4	Any grade	Grade 3 or 4	Any grade	Grade 3 or 4
Total	211 (95.5%)	93 (42.1%)	129 (94.2%)	59 (43.1%)	82 (97.6%)	34 (40.5%)
Anemia	89 (40.3%)	19 (8.6%)	48 (35.0%)	8 (5.8%)	41 (48.8%)	11 (13.1%)
Neutropenia	73 (33.0%)	30 (13.6%)	34 (24.8%)	13 (9.5%)	39 (46.4%)	17 (20.2%)
Thrombocytopenia	64 (29.0%)	27 (12.2%)	33 (24.1%)	14 (10.2%)	31 (36.9%)	13 (15.5%)
Hypoproteinemia	44 (19.9%)	21 (9.5%)	19 (13.9%)	11 (8.0%)	25 (29.8%)	10 (11.9%)
Hypothyroidism	38 (17.2%)	13 (5.9%)	20 (14.6%)	7 (5.1%)	18 (21.4%)	6 (7.1%)
Elevated ALT or AST	34 (15.4%)	7 (3.2%)	21 (15.3%)	4 (2.9%)	13 (15.5%)	3 (3.6%)
Elevated bilirubin	20 (9.0%)	4 (1.8%)	8 (5.8%)	2 (1.5%)	12 (14.3%)	2 (2.4%)
Nausea and vomiting	17 (7.7%)	3 (1.4%)	12 (8.8%)	2 (1.5%)	5 (5.9%)	1 (1.2%)
Rash	14 (6.3%)	4 (1.8%)	6 (4.4%)	1 (0.7%)	8 (9.5%)	3 (3.6%)
Electrolyte disturbance	9 (4.1%)	1 (0.5%)	3 (2.2%)	0	6 (7.1%)	1 (1.2%)
Diarrhea	6 (2.7%)	0	4 (2.9%)	0	2 (2.4%)	0
Fever	5 (2.3%)	0	3 (2.2%)	0	2 (2.4%)	0
Fatigue	4 (1.8%)	1 (0.5%)	3 (2.2%)	0	1 (1.2%)	1 (1.2%)
Allergy	3 (1.4%)	1 (0.5%)	2 (1.4%)	1 (0.7%)	1 (1.2%)	0

ALT, Alanine Aminotransferase; AST, Aspartate Aminotransferase.

A total of 79 patients (35.8%) experienced irAEs, with the most common being hypothyroidism (17.2%), rash (6.3%), and cardiac events (including elevated troponin levels and myocarditis) (5.0%) (**Table 5**). 19 patients (8.6%) experienced grade 3/4 irAEs, including hypothyroidism (13 cases, 5.9%), rash (4 cases, 1.8%), and cardiac events (6 cases, 2.7%). Symptoms of irAEs improved or stabilized with systemic or local corticosteroid therapy and symptomatic supportive care, but 7 patients (3.2%) discontinued treatment due to intolerable irAEs. Among the 12 patients who experienced grade 3/4 irAEs but did not discontinue ICI therapy, all achieved resolution of irAEs after receiving interventions such as corticosteroids. Of these, four patients were identified as having PD during imaging evaluations conducted before the resumption of ICI therapy and therefore did not continue treatment. One patient experienced an allergic reaction upon resuming ICI therapy, which necessitated discontinuation. Another patient voluntarily discontinued treatment due to severe myelosuppression and a poor general condition. One patient developed intolerable rashes after restarting the original ICI regimen and did not proceed with maintenance therapy. Additionally, one patient stopped ICI treatment due to financial constraints. The remaining four patients successfully resumed the original ICI regimen under medical supervision and did not encounter further intolerable irAEs, enabling them to continue treatment. Of the 79 patients with irAEs, 18 received Durvalumab, 10 received Pembrolizumab, 20 received Sintilimab, 10 received Camrelizumab, 4 received Toripalimab, and 17 received other ICIs. Subgroup analysis of patients treated with Durvalumab and Sintilimab showed no significant differences in the incidence of AEs and irAEs between the two groups (p>0.05).

**Table 5:** Immune-Related adverse events.

Immune-related adverse events	All patients (n=221)			First line (n=137)			≥2 lines (n=84)		
	Any grade	Grade 1 or 2	Grade 3 or 4	Any grade	Grade 1 or 2	Grade 3 or 4	Any grade	Grade 1 or 2	Grade 3 or 4
Total	79 (35.8%)	60 (27.1%)	19 (8.6%)	46 (33.6%)	35 (25.5%)	11 (8.0%)	33 (39.3%)	25 (29.8%)	8 (9.5%)
Hypothyroidism	38 (17.2%)	25 (11.3%)	13 (5.9%)	20 (14.6%)	13 (9.5%)	7 (5.1%)	18 (21.4%)	12 (14.3%)	6 (7.1%)
Rash	14 (6.3%)	10 (4.5%)	4 (1.8%)	6 (4.4%)	5 (3.6%)	1 (0.7%)	8 (9.5%)	5 (6.0%)	3 (3.6%)
Cardiac events	11 (5.0%)	5 (2.3%)	6 (2.7%)	5 (3.6%)	2 (1.5%)	3 (2.2%)	6 (7.1%)	3 (3.6%)	3 (3.6%)
Pneumonia	4 (1.8%)	1 (0.5%)	3 (1.4%)	3 (2.2%)	1 (0.7%)	2 (1.5%)	1 (1.2%)	0	1 (1.2%)
Colitis	1 (0.5%)	1 (0.5%)	0	0	0	0	1 (1.2%)	0	0
hepatitis	2 (0.9%)	1(0.5%)	1(0.5%)	1 (0.7%)	1 (0.7%)	0	1 (1.2%)	0	1(1.2%)
Type 1 diabetes	0	0	0	0	0	0	0	0	0
Hypophysitis	0	0	0	0	0	0	0	0	0
Pancreatic events	0	0	0	0	0	0	0	0	0

## 4 Discussion

The treatment options for advanced BTC are limited, and the prognosis is very poor. In recent years, positive results from numerous clinical studies have significantly changed the treatment paradigm for advanced BTC, making ICIs an important therapeutic option for this complex disease (12, 13). However, a substantial amount of real-world research is still needed to provide efficacy and safety data for ICIs in a broader patient population outside of clinical trials. Our study cohort was larger than previous, and the results showed that among the 221 advanced BTC patients treated with ICIs, the ORR and DCR were 23.5% and 74.2%, respectively, with a median PFS and OS of 7.2 months and 12.9 months. 42.1% of patients experienced grade 3/4 AEs, primarily hematological toxicities caused by myelosuppression; 8.6% of patients experienced grade ≥3 irAEs, mainly hypothyroidism and immune-related cardiac events. Our study results demonstrate that ICIs are an effective and safe option for treating advanced BTCs.

We set a minimum criterion of two cycles for ICI infusion, primarily because in China, imaging and efficacy evaluations are typically conducted after two to three cycles of systemic treatment. If tumor progression is observed at this stage, treatment plans are often adjusted, and ICI therapy may be discontinued. Excluding patients who received fewer than four infusions might inadvertently omit a significant subset of individuals who discontinued ICI due to an apparent poor response, potentially leading to a biased representation of the therapy’s impact on advanced BTC. In the entire cohort, 137 patients (62.0%) received ICIs as first-line treatment, with a median OS and PFS of 15.7 months and 8.4 months, respectively, and ORR and DCR of 30.7% and 83.9%. The TOPAZ-1 trial reported that the median OS and PFS in the CisGem combined with Durvalumab group were significantly longer than those in the CisGem combined with the placebo group (median OS 11.5 months vs. 12.8 months, median PFS 5.7 months vs. 7.2 months), with ORR and DCR of 26.7% and 85.3% in the Durvalumab combined treatment group (12). Similarly, the KEYNOTE-966 trial showed that the median OS and PFS for BTC patients treated with pembrolizumab combined with chemotherapy were 12.7 months and 6.5 months, respectively (13). Notably, our first-line cohort achieved superior OS and PFS compared to the TOPAZ-1 and KEYNOTE-966 trials, while the ORR and DCR remained comparable to those observed in TOPAZ-1. The following reasons may explain these longer survival data. Firstly, our study population consisted entirely of Chinese patients, whereas the TOPAZ-1 study observed that the survival period of Asian patients was higher than that of non-Asian patients, with a median OS of up to 13.6 months. The risk of death and progression decreased by 28% and 33%, respectively, and the discontinuation rate due to AEs was lower in the Asian population (29, 30). The specificity of the study population may be one of the important reasons for the long survival observed in our study. Secondly, the TOPAZ-1 study proved that patients with ICC benefited most significantly from Durvalumab combined with CisGem treatment (30). Compared to the TOPAZ-1 study, our first-line treatment included a higher proportion of ICC patients (70.1%) versus 55.3% in TOPAZ-1. Different anatomical sites of tumors in BTC patients respond differently to drugs, and the different proportions of anatomical sites in the study population may be another reason for the better survival data in our study. Thirdly, compared to the fixed treatment protocols in clinical trials, real-world treatment may include a wider variety of treatment combinations, such as intensified chemotherapy regimens, with or without anti-angiogenic therapy, or the addition of radiotherapy or interventional therapy to chemotherapy and immunotherapy. These local treatments not only exert a direct killing effect on tumor cells but may also trigger immunogenic cell death of tumor cells, release tumor-associated antigens, damage-associated molecular patterns (DAMPs), and cytokines that stimulate the host’s immune response while reducing immunosuppressive factors within the tumor (such as regulatory T cells), thereby enhancing the efficacy of ICIs (31). In our first-line ICIs-treated patients, 19.7% received radiotherapy, 25.5% received interventional therapy, and 9.5% received anti-angiogenic therapy. Lastly, in the TOPAZ-1 study, the incidence of AEs was 99.4%, with grade 3/4 AEs at 75.7%. Compared to TOPAZ-1, the incidence of AEs and grade 3/4 AEs in our first-line treatment was lower, at 94.2% and 43.1%, respectively. Therefore, these patient characteristics, heterogeneity in treatment protocols, and the lower incidence of AEs may be the main reasons why our results differ from those of previous clinical studies.

In prior research, Rimini et al. first validated the results of the TOPAZ-1 trial in a real-world setting (18). Their study included 145 patients with advanced BTC receiving Durvalumab in combination with CisGem chemotherapy as first-line treatment, showing a median PFS of 8.9 months and median OS of 12.9 months. Their latest global multicenter real-world study reaffirmed the results of TOPAZ-1. The 666 patients had a median OS of 15.1 months and a median PFS of 8.2 months, which was generally consistent with the survival outcomes of our patients treated with ICIs in the first line (32). However, these studies only explored the performance of single-treatment regimens in real-world scenarios. Another study indicated that PD-1 inhibitor combination therapy in first-line treatment for advanced BTC resulted in a median PFS and OS of 6.6 months and 13.9 months, respectively (33). However, this real-world study had a sample size of only 54 patients, did not include gallbladder cancer patients, and included only PD-1 inhibitors as ICIs. In contrast, 46% of our first-line patients used PD-L1 inhibitors, with higher proportions receiving radiotherapy (19.7% vs. 5.6%) and anti-angiogenic therapy (9.5% vs. 5.6%), potentially contributing to the longer median OS and PFS observed in our study compared to theirs.

The ABC-06 trial investigated the efficacy of FOLFOX regimen as second-line chemotherapy for advanced BTC patients who progressed after first-line treatment (14). Results showed a median OS of only 6.2 months, an ORR of 5.1%, and a 52% incidence of grade 3-5 adverse events. Given its limited survival benefits and high toxicity, there is a need to explore more effective second-line treatment options for advanced BTC. Immune therapy remains investigational in the second-line treatment of advanced BTC. A phase II single-arm clinical trial evaluated 20 advanced BTC patients receiving Sintilimab in combination with anlotinib as second-line therapy, reporting a median OS of 12.3 months, median PFS of 6.5 months, ORR of 30%, and DCR of 95% (34). Another multicenter phase II clinical study demonstrated that nivolumab as salvage therapy for advanced BTC resulted in an ORR of 11%, DCR of 50%, median PFS of 3.68 months, and median OS of 14.24 months (11). Lin et al. explored pembrolizumab in combination with lenvatinib as non-first-line therapy, showing an ORR of 25%, DCR of 78.1%, median PFS of 4.9 months, and median OS of 11.0 months (16). In addition, a real-world study in China included 74 patients who failed gemcitabine-based chemotherapy and received lenvatinib plus PD-1 antibodies as salvage therapy, reporting a median PFS of 4.0 months and median OS of 9.50 months (19). In our study, 84 patients received ICIs as second-line treatment, with a median OS of 9.8 months, median PFS of 5.6 months, ORR of 11.9%, and DCR of 58.3%. Our results show better median OS and ORR compared to ABC-06, likely due to the use of combined local treatment strategies in our salvage therapy patients.

Despite pembrolizumab and Durvalumab being first-line recommended ICIs by CSCO and NCCN guidelines, their high cost and lack of reimbursement in China lead many patients to opt for domestically produced ICIs that are more affordable and accessible. However, there is currently insufficient clinical evidence to establish the efficacy of these domestically produced ICIs in BTC. Unlike pembrolizumab and Durvalumab, which have large phase III Randomized controlled trials (RCTs) like TOPAZ-1 and KEYNOTE-966 supporting their use, domestically produced ICIs have only small single-arm studies as clinical evidence (35–38). For example, a phase II single-arm clinical trial studied Sintilimab in combination with gemcitabine and cisplatin as first-line therapy in 30 patients with advanced BTC, reporting a median OS of 15.9 months, median PFS of 5.1 months, and ORR of 36.7% (37). Another phase II single-arm clinical trial evaluated the efficacy of teraplizumab, lenvatinib in combination with gemcitabine and oxaliplatin (GEMOX) in ICC, showing an ORR of 80% and DCR of 93.3%, though median PFS and OS were not reached (38). A study of camrelizumab in 38 patients with BTC showed an ORR of 54%, median PFS of 6.1 months, and median OS of 11.8 months (35), whereas another study of 47 BTC patients reported an ORR of 7.0% and DCR of 67.4% for camrelizumab plus GEMOX as first-line therapy (36). Due to their small sample sizes, lack of randomization, blinding, control, and maturity of some study indicators, these single-arm studies have limited reliability and low evidence grade. In our study, we compared Durvalumab, a guideline-recommended ICI, with Sintilimab, a domestically produced alternative, in an exploratory analysis of clinical efficacy. Durvalumab showed longer OS and PFS, with no significant differences in ORR or safety profiles. However, Durvalumab patients had better prognostic factors, such as more first-line treatment cases. To address these potential biases, multivariate Cox regression analysis adjusted for these confounders and confirmed Durvalumab’s OS advantage. Nonetheless, previous research suggests that for each covariate included in a regression model, at least 10 events (e.g., deaths) should be observed (26, 27). In our subgroups of Durvalumab and Sintilimab users, the number of patients with outcome events (i.e., deaths) was only 59, and the number of covariates exceeded one-tenth of the effective sample size. To further validate our conclusions, we employed PSM to control for confounding factors. Propensity score methods, first introduced by Rosenbaum and Rubin (25), has become increasingly popular in observational studies for mitigating confounding effects. Among these methods, PSM provides relatively better control over confounders. However, it also has drawbacks, such as excluding unmatched patients, which reduces sample size (24). In our PSM analysis, no significant OS difference was observed, diverging from regression results. This discrepancy likely stems from the exclusion of half of the Sintilimab patients and 17 Durvalumab patients during matching, further diminishing the already limited sample size and reducing statistical power. Moreover, regression analysis results, while statistically significant, carry a heightened risk of Type I errors due to multiple hypothesis testing (e.g., OS, PFS, and ORR comparisons). Adjustments like Bonferroni correction would nullify significance. With a limited sample size and baseline heterogeneity, our findings do not definitively establish Durvalumab’s superiority. However, It is worth noting that achieving statistical significance in small-sample comparisons is challenging, and p-values should be viewed as a reference rather than a conclusive metric. Sole reliance on p-values may underestimate the true clinical significance of the observed difference. Our results suggest a potential advantage of Durvalumab over Sintilimab, highlighting the need for more robust clinical evidence to validate domestically produced ICIs, which are favored in China due to lower cost.

We did not compare other ICIs directly for the following reasons: Both pembrolizumab and Durvalumab are the only ICIs recommended in current guidelines, with robust evidence from large-scale randomized controlled trials supporting their efficacy (13, 30). While no head-to-head comparison exists between the two, our study was not aimed at determining the relative ranking of these ICIs. Instead, our exploratory analysis focused on evaluating the efficacy of domestically produced ICIs, which are more affordable and popular among Chinese patients, and demonstrating the need for further evidence of their efficacy in advanced BTC. As for other domestically produced ICIs, such as camrelizumab, they share similar pricing and accessibility with Sintilimab but also lack sufficient clinical evidence for efficacy in advanced BTC. In our cohort, the number of patients using these ICIs was less than half of those using Sintilimab. Given the sample size limitations already encountered in comparing Sintilimab and Durvalumab, further comparisons involving these other ICIs would inevitably suffer from patient heterogeneity and insufficient sample sizes, precluding meaningful conclusions from regression analyses or PSM. Additionally, conducting multiple pairwise comparisons among these ICIs would further increase the risk of Type I errors under a fixed significance threshold. Our primary aim was to highlight the need for more clinical evidence to validate the efficacy of domestically produced ICIs favored by Chinese patients, rather than ranking these ICIs against guideline-recommended treatments. The comparison between Durvalumab and Sintilimab suffices to meet this objective, and further comparisons with other ICIs are unnecessary at this stage.

With the widespread application of ICIs in the real world, there is an urgent need to identify biomarkers that predict tumor response. Our study identified tumor differentiation, pretreatment CA-199 level<500 U/ml, pretreatment CA-125 level<28.65U/ml, and the number of prior ICIs treatment lines as independent risk factors influencing OS. Factors independently predicting PFS included tumor differentiation, number of ICIs treatment lines, pretreatment CA-199 level, pretreatment CA-125 level and whether subsequent treatments were received. These results aligned with traditional understanding, where poor differentiation and elevated tumor markers were often associated with a worse prognosis. In our multifactorial analysis, NLR did not demonstrate a prognostic effect, despite prior studies showing an association between elevated pretreatment NLR and adverse outcomes in BTC patients (39, 40). This discrepancy may be due to our inclusion of 84 patients who received second-line treatment, often experiencing bone marrow suppression and other adverse effects from prior treatments, which may not reflect the host’s systemic inflammatory status through NLR.

Regarding safety, our study showed that 129 patients (94.2%) experienced any grade of AEs during first-line treatment, with 59 patients (43.1%) experiencing ≥ grade 3 AEs, primarily attributed to chemotherapy-induced neutropenia, anemia, and thrombocytopenia. The incidence rates of overall AEs and grade ≥3 AEs in our study were comparable to other real-world studies (18, 33) but lower than those reported in the TOPAZ-1 trial (99.4% and 75.7%, respectively) (30) and the KEYNOTE-966 trial (99% and 79%, respectively) (13), potentially due to enhanced monitoring practices in RCTs compared to real-world settings. Forty-six patients (33.6%) experienced irAEs, with a rate of grade 3-4 irAEs at 8.0%. These results are similar to some real-world findings (33) but higher than those reported in TOPAZ-1 (12.7% and 2.4%, respectively) (30) and KEYNOTE-966 (22% and 7%, respectively) (13). Several reasons might explain these discrepancies. First, our study included patients receiving later-line treatments, who might have experienced cumulative toxicity. Second, our analysis involved multiple types of ICIs, and prior studies have indicated that PD-1 inhibitors are associated with higher irAE rates than PD-L1 inhibitors (41), potentially explaining why our data align more closely with KEYNOTE-966 and are higher than TOPAZ-1. Additionally, patients with autoimmune diseases are typically excluded from RCTs, yet previous studies have indicated that these patients may have a higher incidence of irAEs (42, 43). In second-line treatment, the incidence rates of AEs and ≥ grade 3 AEs were 97.6% and 40.5%, respectively, consistent with previous real-world study data (19).

Our study has several limitations. Firstly, its retrospective design may introduce selection bias, particularly concerning treatment allocation. Secondly, as the study was conducted at a single medical center, the generalizability of the findings may be limited, highlighting the need for multicenter validation. Thirdly, the relatively small sample size in subgroup analyses could limit statistical power. Additionally, the inclusion of different treatment regimens, such as chemotherapy or anti-angiogenic therapy, may affect the interpretation of ICI-related outcomes. Lastly, the lack of biomarker data (e.g., PD-L1 expression, MSI status) in our study precluded the analysis of predictive factors.

## 5 Conclusion

This real-world study demonstrates that ICIs provide clinically meaningful survival benefits in both first-line (median OS 15.7 months) and ≥2nd-line settings (median OS 9.8 months) for advanced BTC. Notably, in addition to the first-line treatment commonly investigated by most studies, our data represent a large real-world cohort validating ICIs in later-line settings, where outcomes have historically been dismal (e.g., ABC-06 OS 6.2 months with chemotherapy alone). While domestically produced ICIs are widely used in clinical practice in China, observed differences in efficacy (unadjusted HR 2.47 for OS comparing Sintilimab with Durvalumab) and the lack of robust clinical trial evidence underscore the need for further validation. These findings underscore the importance of real-world data in complementing trial evidence for BTC treatment optimization.

## Data Availability

The datasets presented in this article are not readily available because data supporting the results of this research can be reasonably requested from the corresponding authors. Requests to access the datasets should be directed to maji@wchscu.cn.
