# Supplementary material for: Efficacy and safety of immune checkpoint inhibitors in advanced biliary tract cancer: a real-world study
Source: Front Immunol. 2025 Mar 31;16:1493234. doi: 10.3389/fimmu.2025.1493234 (PMC11994646; doi:10.3389/fimmu.2025.1493234)
Supplement: Supplementary file 1 [file DataSheet1.docx]

Supplementary Material

# Supplementary Figures and Tables

## Supplementary Figures


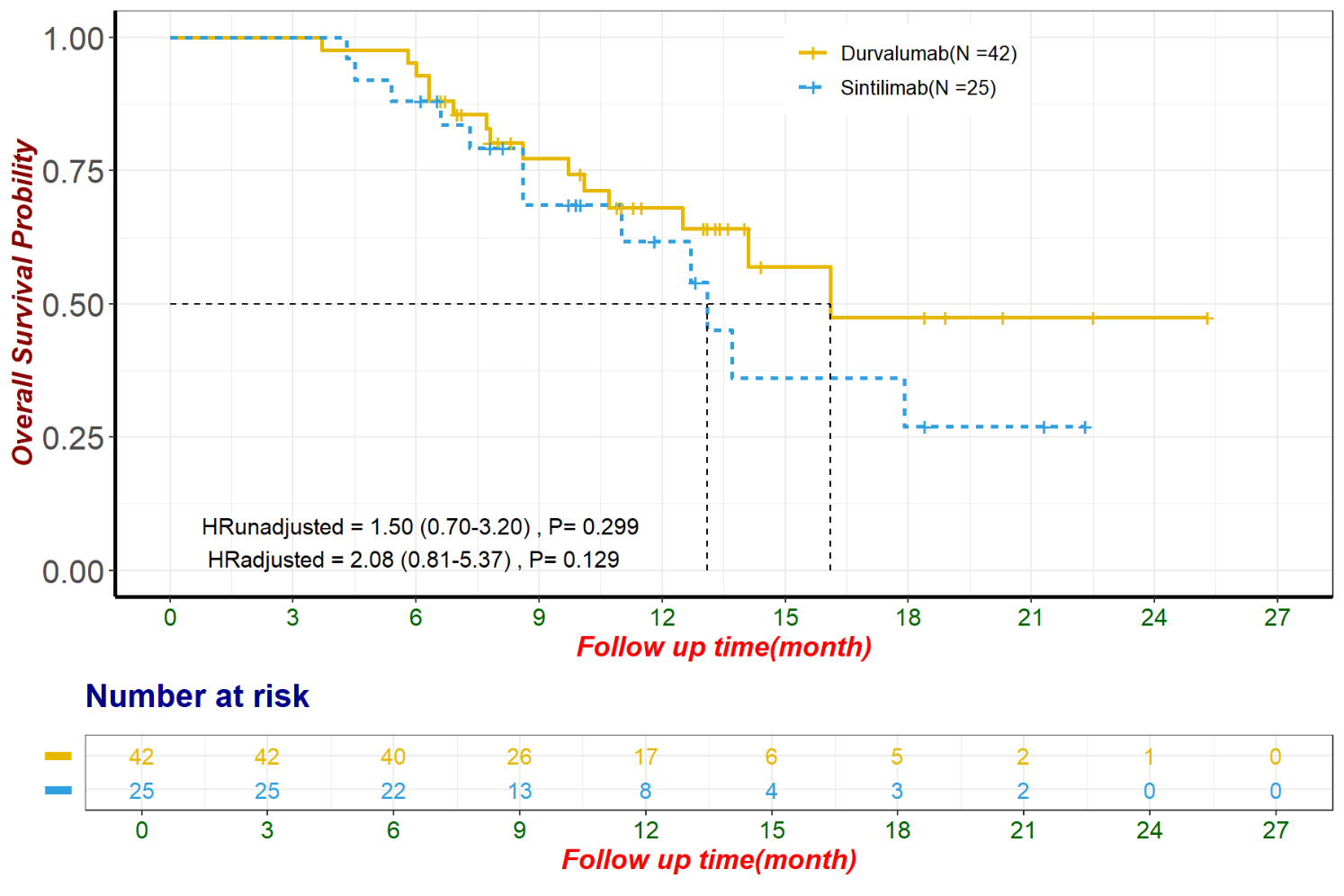


**Supplementary Figure 1.** Kaplan-Meier curve of Overall Survival in patients using Durvalumab and Sintilimab after propensity score matching. HRunadjusted and HRadjusted represent the hazard ratios for Sintilimab compared to Durvalumab, derived from univariate and multivariate Cox proportional hazards regression analyses, respectively.


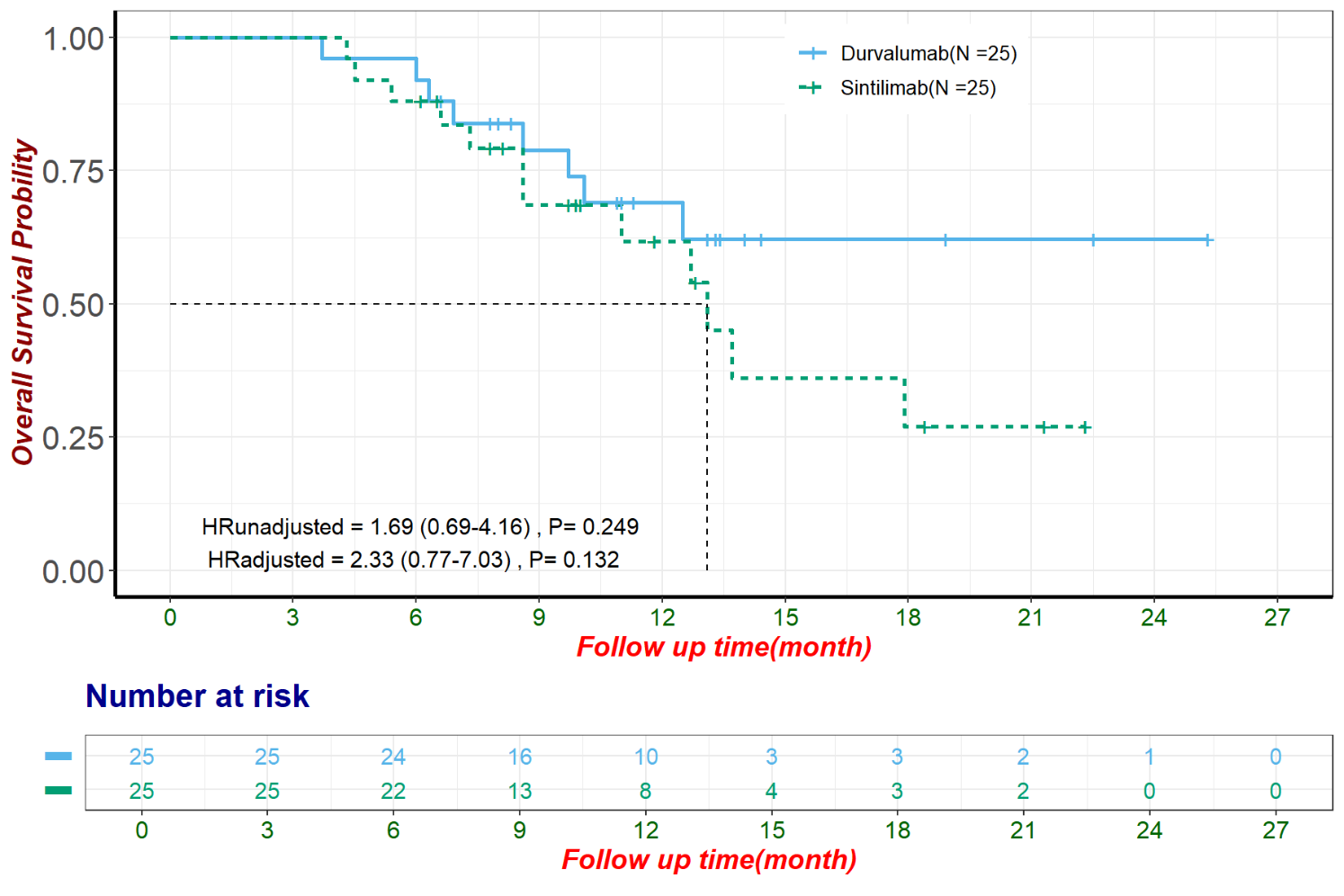


**Supplementary Figure 2.** Kaplan-Meier curve of overall survival in patients treated with Durvalumab versus Sintilimab after propensity score matching (1:1 matching ratio) as a sensitivity analysis. HRunadjusted and HRadjusted represent the hazard ratios for Sintilimab compared to Durvalumab, derived from univariate and multivariate Cox proportional hazards regression analyses, respectively.

## Supplementary Tables

| **Supplementary Table 1: Univariate and Multivariate Cox Proportional Hazards Regression for PFS in the Overall Population** | | | |
| --- | --- | --- | --- |
| **Covariate** | **All patients (n=221)** | **HR (univariable)** | **HR (multivariable)** |
| Sex |  |  |  |
| Male | 117 (52.9%) |  |  |
| Female | 104 (47.1%) | 1.02 (0.76-1.38, p=.888) |  |
| Age (Mean ± SD) | 58.0 ± 10.3 | 1.01 (0.99-1.02, p=.445) |  |
| Virology status |  |  |  |
| No viral hepatitis | 68 (30.8%) |  |  |
| Any viral hepatitis B | 152 (68.8%) | 1.06 (0.76-1.47, p=.736) |  |
| Prior hepatitis C | 1 (0.5%) | 1.34 (0.18-9.71, p=.773) |  |
| Disease status |  |  |  |
| Initially unresectable | 110 (49.8%) |  |  |
| Recurrent | 111 (50.2%) | 1.17 (0.87-1.58, p=.309) |  |
| Disease classification |  |  |  |
| Locally advanced | 44 (19.9%) |  |  |
| Metastatic | 177 (80.1%) | 1.00 (0.68-1.48, p=.983) |  |
| Site of origin |  |  |  |
| Intrahepatic | 142 (64.3%) |  |  |
| Extrahepatic | 41 (18.6%) | 1.09 (0.73-1.61, p=.676) | 1.12 (0.74-1.69, p=.585) |
| Gallbladder | 38 (17.2%) | 1.53 (1.05-2.24, p=.028)* | 1.08 (0.71-1.63, p=.717) |
| Degree of differentiation |  |  |  |
| Poorly | 137 (62.0%) |  |  |
| Moderately-to-well | 84 (38.0%) | 0.73 (0.53-0.99, p=.044)* | 0.59 (0.43-0.82, p=.002)* |
| Type of ICIs |  |  |  |
| Anti-PD-1 | 148 (67.0%) |  |  |
| Anti-PD-L1 | 73 (33.0%) | 0.68 (0.49-0.95, p=.023)* | 0.84 (0.58-1.20, p=.339) |
| Combination with chemotherapy |  |  |  |
| No | 29 (13.1%) |  |  |
| Yes | 192 (86.9%) | 0.65 (0.43-0.97, p=.036)* | 1.14 (0.71-1.84, p=.585) |
| Combination with anti-angiogenic drugs |  |  |  |
| No | 180 (81.4%) |  |  |
| Yes | 41 (18.6%) | 1.10 (0.77-1.59, p=.599) |  |
| Line of treatment for ICIs |  |  |  |
| First line | 137 (62.0%) |  |  |
| ≥2 lines | 84 (38.0%) | 1.86 (1.38-2.52, p<.001)* | 1.66 (1.16-2.38, p=.006)* |
| ECOG performance status |  |  |  |
| 0 | 165 (74.7%) |  |  |
| ≥1 | 56 (25.3%) | 0.76 (0.54-1.08, p=.127) |  |
| Have received radiotherapy |  |  |  |
| No | 168 (76.0%) |  |  |
| Yes | 53 (24.0%) | 0.87 (0.62-1.23, p=.436) |  |
| Have undergone interventional therapy |  |  |  |
| No | 165 (74.7%) |  |  |
| Yes | 56 (25.3%) | 1.00 (0.71-1.40, p=.988) |  |
| Occurrence of irAE |  |  |  |
| No | 142 (64.3%) |  |  |
| Yes | 79 (35.7%) | 0.87 (0.63-1.19, p=.375) |  |
| Pre-treatment CA199 level less than 500 U/mL |  |  |  |
| No | 65 (29.4%) |  |  |
| Yes | 156 (70.6%) | 0.55 (0.40-0.76, p<.001)* | 0.52 (0.37-0.72, p<.001)* |
| Pre-treatment CEA level less than 5 ng/mL |  |  |  |
| No | 82 (37.1%) |  |  |
| Yes | 139 (62.9%) | 0.69 (0.51-0.94, p=.018)* | 0.91 (0.64-1.31, p=.620) |
| Pre-treatment CA125 less than 28.65 U/mL |  |  |  |
| No | 115 (52.0%) |  |  |
| Yes | 106 (48.0%) | 0.60 (0.44-0.82, p=.001)* | 0.69 (0.49-0.98, p=.036)* |
| NLR less than 3 |  |  |  |
| No | 110 (49.8%) |  |  |
| Yes | 111 (50.2%) | 0.91 (0.67-1.23, p=.531) |  |
| Received subsequent treatment |  |  |  |
| No | 124 (56.1%) |  |  |
| Yes | 97 (43.9%) | 2.82 (2.07-3.83, p<.001)* | 2.79 (2.03-3.84, p<.001)* |
| Use of antibiotics within one month after immunization |  |  |  |
| No | 211 (95.5%) |  |  |
| Yes | 10 (4.5%) | 1.11 (0.52-2.36, p=.792) |  |
| Smoking status |  |  |  |
| Never | 170 (76.9%) |  |  |
| Former/Current | 51 (23.1%) | 0.88 (0.62-1.25, p=.463) |  |
| n=221 events=174 (for PFS, events refer to disease progression or death, whichever occurred first)  CA125, Cancer Antigen 125; CA199, Carbohydrate Antigen 19-9; CEA, Carcinoembryonic Antigen; ECOG, Eastern Cooperative Oncology Group; ICIs, Immune Checkpoint Inhibitors; irAE, immune-related Adverse Event; NLR, Neutrophil-to-Lymphocyte Ratio; PD-1, Programmed Death-1; PD-L1, Programmed Death-Ligand 1; PFS, progression-free survival  **P*<0.05 | | | |

| **Supplementary Table 2: Baseline Characteristics Before Propensity Score Matching for Patients Using Durvalumab and Sintilimab** | | | | |
| --- | --- | --- | --- | --- |
| **Characteristic** | **Overall N = 109***^1^* | **Durvalumab N = 59***^1^* | **Sintilimab N = 50***^1^* | **p-value***^2^* |
| Age years | 58 (11) | 60 (12) | 56 (8) | 0.015* |
| Sex |  |  |  | 0.5 |
| Male | 55 (50%) | 28 (47%) | 27 (54%) |  |
| Female | 54 (50%) | 31 (53%) | 23 (46%) |  |
| Virology_status |  |  |  | 0.2 |
| No viral hepatitis | 33 (30%) | 21 (36%) | 12 (24%) |  |
| Any viral hepatitis B | 76 (70%) | 38 (64%) | 38 (76%) |  |
| Prior hepatitis C | 0 (0%) | 0 (0%) | 0 (0%) |  |
| Disease_status |  |  |  | <0.001* |
| Initially unresectable | 53 (49%) | 38 (64%) | 15 (30%) |  |
| Recurrent | 56 (51%) | 21 (36%) | 35 (70%) |  |
| Disease_classification |  |  |  | 0.7 |
| Locally advanced | 17 (16%) | 10 (17%) | 7 (14%) |  |
| Metastatic | 92 (84%) | 49 (83%) | 43 (86%) |  |
| Site_of_origin |  |  |  | 0.2 |
| Intrahepatic | 72 (66%) | 43 (73%) | 29 (58%) |  |
| Extrahepatic | 19 (17%) | 9 (15%) | 10 (20%) |  |
| Gallbladder | 18 (17%) | 7 (12%) | 11 (22%) |  |
| Degree_of_differentiation |  |  |  | 0.4 |
| Poorly | 68 (62%) | 39 (66%) | 29 (58%) |  |
| Moderately-to-well | 41 (38%) | 20 (34%) | 21 (42%) |  |
| Combination_with_chemotherapy |  |  |  | 0.003* |
| Yes | 102 (94%) | 59 (100%) | 43 (86%) |  |
| No | 7 (6%) | 0 (0%) | 7 (14%) |  |
| Combination_with_anti_angiogenic_drugs |  |  |  | <0.001* |
| Yes | 15 (14%) | 2 (3.4%) | 13 (26%) |  |
| No | 94 (86%) | 57 (96.6%) | 37 (74%) |  |
| ECOG_performance_status |  |  |  | 0.8 |
| 0 | 80 (73%) | 44 (75%) | 36 (72%) |  |
| ≥1 | 29 (27%) | 15 (25%) | 14 (28%) |  |
| Have_received_radiotherapy |  |  |  | 0.9 |
| Yes | 29 (27%) | 16 (27%) | 13 (26%) |  |
| No | 80 (73%) | 43 (73%) | 37 (74%) |  |
| Have_undergone_interventional_therapy |  |  |  | 0.2 |
| Yes | 29 (27%) | 13 (22%) | 16 (32%) |  |
| No | 80 (73%) | 46 (78%) | 34 (68%) |  |
| Pre_treatment_CA199_level_less_than_500 U/mL |  |  |  | 0.7 |
| Yes | 74 (68%) | 41 (69%) | 33 (66%) |  |
| No | 35 (32%) | 18 (31%) | 17 (34%) |  |
| Pre_treatment_CEA_level_less_than_5 ng/mL |  |  |  | 0.5 |
| Yes | 71 (65%) | 40 (68%) | 31 (62%) |  |
| No | 38 (35%) | 19 (32%) | 19 (38%) |  |
| Pre_treatment_CA125_less_than_28.65 U/mL |  |  |  | 0.029* |
| Yes | 56 (51%) | 36 (61%) | 20 (40%) |  |
| No | 53 (49%) | 23 (39%) | 30 (60%) |  |
| NLR_less_than_3 |  |  |  | 0.076 |
| Yes | 58 (53%) | 36 (61%) | 22 (44%) |  |
| No | 51 (47%) | 23 (39%) | 28 (56%) |  |
| Line_of_treatment_for_ICIs |  |  |  | <0.001* |
| First line | 73 (67%) | 52 (88%) | 21 (42%) |  |
| ≥2 lines | 36 (33%) | 7 (12%) | 29 (58%) |  |
| Received_subsequent_treatment |  |  |  | 0.5 |
| Yes | 50 (46%) | 29 (49%) | 21 (42%) |  |
| No | 59 (54%) | 30 (51%) | 29 (58%) |  |
| Use_of_antibiotics_within_one_month_after_immunization |  |  |  | >0.9 |
| Yes | 4 (3.7%) | 2 (3.4%) | 2 (4.0%) |  |
| No | 105 (96.3%) | 57 (96.6%) | 48 (96.0%) |  |
| Smoking_status |  |  |  | >0.9 |
| Never | 83 (76%) | 45 (76%) | 38 (76%) |  |
| Former/Current | 26 (24%) | 14 (24%) | 12 (24%) |  |
| CA125, Cancer Antigen 125; CA199, Carbohydrate Antigen 19-9; CEA, Carcinoembryonic Antigen; ECOG, Eastern Cooperative Oncology Group; ICIs, Immune Checkpoint Inhibitors; NLR, Neutrophil-to-Lymphocyte Ratio  *^1^* Sample mean (SD), for age characteristic; Sample size n (percent %), for others  *^2^* Wilcoxon rank sum test; Pearson’s Chi-squared test; Fisher’s exact test  **P*<0.05 | | | | |

| **Supplementary Table 3: Univariate and Multivariate Cox Proportional Hazards Regression for OS in the Unmatched Durvalumab and Sintilimab Cohorts** | | | |
| --- | --- | --- | --- |
| **Covariate** | **All patients (n=109)** | **HR (univariable)** | **HR (multivariable)** |
| Sex |  |  |  |
| Male | 55 (50.5%) |  |  |
| Female | 54 (49.5%) | 0.99 (0.59-1.65, p=.969) |  |
| Age (Mean ± SD) | 58.1 ± 10.9 | 1.00 (0.98-1.02, p=.830) |  |
| Virology status |  |  |  |
| No viral hepatitis | 33 (30.3%) |  |  |
| Any viral hepatitis B | 76 (69.7%) | 1.24 (0.70-2.18, p=.462) |  |
| Disease status |  |  |  |
| Initially unresectable | 53 (48.6%) |  |  |
| Recurrent | 56 (51.4%) | 0.99 (0.59-1.65, p=.957) |  |
| Disease classification |  |  |  |
| Locally advanced | 17 (15.6%) |  |  |
| Metastatic | 92 (84.4%) | 1.58 (0.74-3.35, p=.236) |  |
| Site of origin |  |  |  |
| Intrahepatic | 72 (66.1%) |  |  |
| Extrahepatic | 19 (17.4%) | 1.54 (0.79-2.99, p=.204) | 1.11 (0.54-2.29, p=.775) |
| Gallbladder | 18 (16.5%) | 2.21 (1.19-4.11, p=.012)* | 1.03 (0.51-2.10, p=.924) |
| Degree of differentiation |  |  |  |
| Poorly | 68 (62.4%) |  |  |
| Moderately-to-well | 41 (37.6%) | 0.50 (0.29-0.86, p=.013)* | 0.29 (0.15-0.59, p=.001)* |
| ICI |  |  |  |
| Durvalumab | 59 (54.1%) |  |  |
| Sintilimab | 50 (45.9%) | 2.47 (1.46-4.20, p<.001)* | 2.16 (1.07-4.36, p=.031)* |
| Combination with chemotherapy |  |  |  |
| No | 7 (6.4%) |  |  |
| Yes | 102 (93.6%) | 0.39 (0.18-0.88, p=.022)* | 0.90 (0.32-2.51, p=.834) |
| Combination with anti-angiogenic drugs |  |  |  |
| No | 94 (86.2%) |  |  |
| Yes | 15 (13.8%) | 1.31 (0.66-2.59, p=.437) |  |
| Line of treatment for ICIs |  |  |  |
| First line | 73 (67.0%) |  |  |
| ≥2 lines | 36 (33.0%) | 1.73 (1.04-2.90, p=.036)* | 1.50 (0.74-3.05, p=.259) |
| ECOG performance status |  |  |  |
| 0 | 80 (73.4%) |  |  |
| ≥1 | 29 (26.6%) | 1.64 (0.95-2.84, p=.077) | 2.17 (1.12-4.19, p=.022)* |
| Have received radiotherapy |  |  |  |
| No | 80 (73.4%) |  |  |
| Yes | 29 (26.6%) | 1.12 (0.63-1.97, p=.703) |  |
| Have undergone interventional therapy |  |  |  |
| No | 80 (73.4%) |  |  |
| Yes | 29 (26.6%) | 0.86 (0.49-1.54, p=.619) |  |
| Occurrence of irAE |  |  |  |
| No | 71 (65.1%) |  |  |
| Yes | 38 (34.9%) | 0.98 (0.57-1.69, p=.948) |  |
| Pre-treatment CA199 level less than 500 U/mL |  |  |  |
| No | 35 (32.1%) |  |  |
| Yes | 74 (67.9%) | 0.51 (0.30-0.86, p=.012)* | 0.39 (0.21-0.75, p=.005)* |
| Pre-treatment CEA level less than 5 ng/mL |  |  |  |
| No | 38 (34.9%) |  |  |
| Yes | 71 (65.1%) | 0.31 (0.18-0.53, p<.001)* | 0.37 (0.18-0.75, p=.006)* |
| Pre-treatment CA125 less than 28.65 U/mL |  |  |  |
| No | 53 (48.6%) |  |  |
| Yes | 56 (51.4%) | 0.37 (0.22-0.64, p<.001)* | 0.86 (0.41-1.80, p=.692) |
| NLR less than 3 |  |  |  |
| No | 51 (46.8%) |  |  |
| Yes | 58 (53.2%) | 0.53 (0.31-0.89, p=.016)* | 0.65 (0.35-1.21, p=.178) |
| Received subsequent treatment |  |  |  |
| No | 59 (54.1%) |  |  |
| Yes | 50 (45.9%) | 1.19 (0.71-2.00, p=.503) |  |
| Use of antibiotics within one month after immunization |  |  |  |
| No | 105 (96.3%) |  |  |
| Yes | 4 (3.7%) | 3.50 (1.25-9.78, p=.017)* | 4.93 (1.45-16.81, p=.011)* |
| Smoking status |  |  |  |
| Never | 83 (76.1%) |  |  |
| Former/Current | 26 (23.9%) | 0.84 (0.45-1.59, p=.594) |  |
| n=109, events=59 (for OS, events refer to the number of deaths caused by cancer)  CA125, Cancer Antigen 125; CA199, Carbohydrate Antigen 19-9; CEA, Carcinoembryonic Antigen; ECOG, Eastern Cooperative Oncology Group; ICIs, Immune Checkpoint Inhibitors; irAE, immune-related Adverse Event; NLR, Neutrophil-to-Lymphocyte Ratio; OS, Overall Survival; PD-1, Programmed Death-1; PD-L1, Programmed Death-Ligand 1  **P*<0.05 | | | |

| **Supplementary Table 4: Baseline Characteristics After Propensity Score Matching for Populations Using Durvalumab and Sintilimab** | | | | |
| --- | --- | --- | --- | --- |
| **Characteristic** | **Overall, N = 67***^1^* | **Durvalumab N = 42***^1^* | **Sintilimab, N = 25***^1^* | **p-value***^2^* |
| Age, years | 59 (11) | 60 (12) | 56 (8) | 0.03* |
| Sex |  |  |  | 0.4 |
| Male | 36 (54%) | 21 (50%) | 15 (60%) |  |
| Female | 31 (46%) | 21 (50%) | 10 (40%) |  |
| Virology_status |  |  |  | 0.07 |
| No viral hepatitis | 19 (28%) | 15 (36%) | 4 (16%) |  |
| Any viral hepatitis B | 48 (72%) | 27 (64%) | 21 (84%) |  |
| Disease_status |  |  |  | 0.001* |
| Initially unresectable | 36 (54%) | 29 (69%) | 7 (28%) |  |
| Recurrent | 31 (46%) | 13 (31%) | 18 (72%) |  |
| Disease_classification |  |  |  | 0.7 |
| Locally advanced | 11 (16%) | 6 (14%) | 5 (19%) |  |
| Metastatic | 56 (84%) | 36 (86%) | 20 (80%) |  |
| Site_of_origin |  |  |  | 0.7 |
| Intrahepatic | 47 (70%) | 30 (71%) | 17 (68%) |  |
| Extrahepatic | 13 (19%) | 7 (17%) | 6 (24%) |  |
| Gallbladder | 7 (10%) | 5 (12%) | 2 (8%) |  |
| Degree_of_differentiation |  |  |  | 0.3 |
| Poorly | 40 (60%) | 27 (64%) | 13 (52%) |  |
| moderately-to-well | 27 (40%) | 15 (36%) | 12 (48%) |  |
| Combination_with_chemotherapy |  |  |  | >0.9 |
| No | 0 | 0 | 0 |  |
| Yes | 67 (100%) | 42 (100%) | 25 (100%) |  |
| Combination_with_anti_angiogenic_drugs |  |  |  | 0.02* |
| No | 61 (91%) | 41 (98%) | 20 (80%) |  |
| Yes | 6 (9%) | 1 (2%) | 5 (20%) |  |
| ECOG_performance_status |  |  |  | 0.5 |
| 0 | 51 (76%) | 33 (79%) | 18 (72%) |  |
| 1 | 16 (24%) | 9 (21%) | 7 (28%) |  |
| Have_received_radiotherapy |  |  |  | 0.3 |
| No | 52 (78%) | 31 (74%) | 21 (84%) |  |
| Yes | 15 (22%) | 11 (26%) | 4 (16%) |  |
| Have_undergone_interventional_therapy |  |  |  | 0.1 |
| No | 48 (72%) | 33 (79%) | 15 (60%) |  |
| Yes | 19 (28%) | 9 (21%) | 10 (40%) |  |
| Pre_treatment_CA199_level_less_than_500 U/mL |  |  |  | 0.3 |
| No | 22 (33%) | 12 (29%) | 10 (40%) |  |
| Yes | 45 (67%) | 30 (71%) | 15 (60%) |  |
| Pre_treatment_CEA_level_less_than_5 ng/mL |  |  |  | 0.9 |
| No | 18 (27%) | 11 (26%) | 7 (28%) |  |
| Yes | 49 (73%) | 31 (74%) | 18 (72%) |  |
| Pre_treatment_CA125_less_than_28.65 U/mL |  |  |  | >0.9 |
| No | 29 (43%) | 18 (43%) | 11 (44%) |  |
| Yes | 38 (57%) | 24 (57%) | 14 (56%) |  |
| NLR_less_than_3 |  |  |  | >0.9 |
| No | 29 (43%) | 18 (43%) | 11 (44%) |  |
| Yes | 38 (57%) | 24 (57%) | 14 (56%) |  |
| Line_of_treatment_for_ICIs |  |  |  | 0.7 |
| First line | 56 (84%) | 36 (86%) | 20 (80%) |  |
| ≥2 lines | 11 (16%) | 6 (14%) | 5 (20%) |  |
| Received_subsequent_treatment |  |  |  | 0.2 |
| No | 34 (51%) | 19 (45%) | 15 (60%) |  |
| Yes | 33 (49%) | 23 (55%) | 10 (40%) |  |
| Use_of_antibiotics_within_one_month_after_immunization |  |  |  | 0.6 |
| No | 63 (94%) | 40 (95%) | 23 (92%) |  |
| Yes | 4 (6%) | 2 (5%) | 2 (8%) |  |
| Smoking_status |  |  |  | >0.9 |
| Never | 46 (69%) | 29 (69%) | 17 (68%) |  |
| Former/Current | 21 (31%) | 13 (31%) | 8 (32%) |  |
| CA125, Cancer Antigen 125; CA199, Carbohydrate Antigen 19-9; CEA, Carcinoembryonic Antigen; ECOG, Eastern Cooperative Oncology Group; ICIs, Immune Checkpoint Inhibitors; NLR, Neutrophil-to-Lymphocyte Ratio  *^1^* Sample mean (SD), for age characteristic; Sample size n (percent %), for others  *^2^* Wilcoxon rank sum test; Pearson’s Chi-squared test; Fisher’s exact test  **P*<0.05 | | | | |
